# Vitamin D status and outcomes for hospitalised older patients with COVID-19

**DOI:** 10.1136/postgradmedj-2020-138712

**Published:** 2020-08-27

**Authors:** Vadir Baktash, Tom Hosack, Nishil Patel, Shital Shah, Pirabakaran Kandiah, Koenraad Van den Abbeele, Amit K J Mandal, Constantinos G Missouris

**Affiliations:** Department of Medicine, Frimley Health NHS Foundation Trust, Wexham Park Hospital, Slough, UK; Department of Medicine, Frimley Health NHS Foundation Trust, Wexham Park Hospital, Slough, UK; Department of Medicine, Frimley Health NHS Foundation Trust, Wexham Park Hospital, Slough, UK; Department of Medicine, Frimley Health NHS Foundation Trust, Wexham Park Hospital, Slough, UK; Department of Medicine, Frimley Health NHS Foundation Trust, Wexham Park Hospital, Slough, UK; Department of Medicine, Frimley Health NHS Foundation Trust, Wexham Park Hospital, Slough, UK; Department of Medicine, Frimley Health NHS Foundation Trust, Wexham Park Hospital, Slough, UK; Department of Medicine, Frimley Health NHS Foundation Trust, Wexham Park Hospital, Slough, UK; Department of Cardiology, University of Cyprus Medical School, Nicosia, Cyprus

**Keywords:** General medicine (see internal medicine), geriatric medicine, diabetes & endocrinology, calcium & bone

## Abstract

**Purpose:**

Older adults are more likely to be vitamin D deficient. The aim of the study was to determine whether these patients have worse outcomes with COVID-19.

**Methods:**

We conducted a prospective cohort study between 1 March and 30 April 2020 to assess the importance of vitamin D deficiency in older patients with COVID-19. The cohort consisted of patients aged ≥65 years presenting with symptoms consistent with COVID-19 (n=105). All patients were tested for serum 25-hydroxyvitamin D (25(OH)D) levels during acute illness. Diagnosis of COVID-19 was confirmed via viral reverse transcriptase PCR swab or supporting radiological evidence. COVID-19-positive arm (n=70) was sub-divided into vitamin D-deficient (≤30 nmol/L) (n=39) and -replete groups (n=35). Subgroups were assessed for disease severity using biochemical, radiological and clinical markers. Primary outcome was in-hospital mortality. Secondary outcomes were laboratory features of cytokine storm, thoracic imaging changes and requirement of non-invasive ventilation (NIV).

**Results:**

COVID-19-positive arm demonstrated lower median serum 25(OH)D level of 27 nmol/L (IQR=20–47 nmol/L) compared with COVID-19-negative arm, with median level of 52 nmol/L (IQR=31.5–71.5 nmol/L) (p value=0.0008). Among patients with vitamin D deficiency, there was higher peak D-dimer level (1914.00 μgFEU/L vs 1268.00 μgFEU/L) (p=0.034) and higher incidence of NIV support and high dependency unit admission (30.77% vs 9.68%) (p=0.042). No increased mortality was observed between groups.

**Conclusion:**

Older adults with vitamin D deficiency and COVID-19 may demonstrate worse morbidity outcomes. Vitamin D status may be a useful prognosticator.

## INTRODUCTION

The COVID-19 outbreak, which began in China in late 2019 and then rapidly spread across the world, has spurred a global effort to tackle the disease and establish risk factors and prognostic markers; one such is serum vitamin D deficiency. Vitamin D is a secosteroid with varied immunomodulatory, anti-inflammatory, antifibrotic and antioxidant actions. There is growing evidence that it may play a role in the pathophysiological processes of COVID-19.

The relationship between vitamin D deficiency and adverse prognosis has been suggested by the apparent Northern–Southern latitude gradient, with mortality and hospitalisation rates for COVID-19 seen to be higher in northern latitude countries compared with those closer to the equator.^1^ Furthermore, research by Alipio and colleagues^2^ , in a retrospective study, provides evidence of an association between vitamin D deficiency and adverse outcome in patients with COVID-19. Older adults in institutions, such as hospitals and care homes, are particularly likely to be vitamin D deficient as a result of a lack of sun exposure and dietary insufficiency, and may have worse outcomes with COVID-19.

In the UK, The Royal College of Physicians of London Commentary^3^ reported that patients who died of COVID-19 were severely vitamin D deficient. There is also growing concern that the Black, Asian and Minority Ethnic community who produce less vitamin D as a result of higher skin melanin content are inherently more susceptible to severe presentations of COVID-19.^4^ Therefore, the British Nutrition Found^2^ation,^5^ who applied guidance to the public amidst self-isolation for COVID-19, has suggested that everyone should consider taking a daily supplement of 400 IU of vitamin D.

We investigated serum vitamin D levels in older patients admitted to our institution during the pandemic. The aim of our study was to assess the potential relationship between vitamin D deficiency and COVID-19 severity in hospitalised older adults. We conducted a single-centred prospective cohort study of older patients admitted to a large district general hospital in the UK, with symptoms in keeping with a viral infection. Patients were divided into COVID-19-positive and -negative groups with subsequent assessment of vitamin D status. A subgroup analysis of the COVID-19-positive arm was conducted with analysis of serum markers of infection and clinical markers of disease severity, such as high dependency unit (HDU) admission and non-invasive ventilation (NIV).

## METHODS AND MATERIALS

### Population

**Figure 1:**
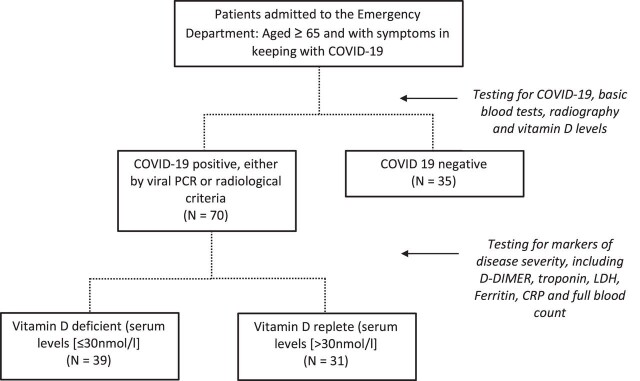
Flow chart, depicting recruitment of patients upon admission via the emergency department, with subsequent separation. CRP, C reactive protein; LDH, lactate dehydrogenase.

All emergency admissions aged ≥65 years admitted to our hospital with symptoms consistent with COVID-19 including cough, dyspnoea, fever and/or anosmia^6^ between 1 March and 30 April 2020 were included in the study (figure 1). These patients were investigated with real-time reverse transcriptase-PCR (RT-PCR) assay for severe acute respiratory syndrome coronavirus-2 (SARS-CoV-2) on nasopharyngeal swab, blood tests including vitamin D levels (25-hydroxyvitamin D (25(OH)D), basic observations, and chest X-ray (CXR) and/or chest CT.

Diagnosis of active SARS-CoV-2 infection was based on positive viral RT-PCR swab or evidence of COVID-19 on a chest radiograph or chest CT (bilateral peripheral infiltrates/ground-glass opacities, airspace opacification, traction bronchiectasis, inter/intralobular septal thickening and organising pneumonia). Patients who did not meet either of these criteria were enrolled into the COVID-19-negative group.

The COVID-19-positive group was divided into vitamin D-deficient (≤30 nmol/L) and -replete (>30 nmol/L) groups, as per national guidelines and local laboratory standards.^7^ Vitamin D-deficient patients were supplemented in accordance with national guidelines and all patients received care in line with best practice guidance. In addition, patients were treated with subcutaneous low-molecular-weight heparin venous thromboembolism (VTE) prophylaxis as per national guidance.

### Data collection

Data were extracted from medical notes and the local hospital electronic database. These included age, weight, height, ethnicity, smoking status and comorbidities. Ethnicities were self-assigned. Rockwood Clinical Frailty Score^8^ and Charlson Comorbidity Index^9^ were calculated retrospectively.

The primary outcome measured was in-hospital mortality secondary to COVID-19. Secondary outcomes were defined as NIV support and admission to HDU, COVID-19 radiographic changes on CXR and laboratory features of cytokine storm. All patients had vitamin D levels checked in keeping with good medical practice. Biochemical/haematological panels (C reactive protein (CRP), D-dimer, ferritin, high sensitivity troponin T, lactate dehydrogenase (LDH) and lymphocyte count) were carried out in accordance with local guidance on COVID-19 diagnostics and prognostication.^10^ In addition, data for specific features consistent with the COVID-19^11 12^ were collected from formal reports of CXR and/or chest CT. Causes of death were obtained digitally from mortality reports made by our hospital’s mortality and bereavement office.

### Statistical analysis

All statistical analyses were carried out on GraphPad Prism (version 8). For continuous outcome variables, each data set was assessed for normality using Kolmogorov-Smirnov and Shapiro-Wilk tests, and tested for significance with either an unpaired t-test if parametric or a Mann-Whitney test if non-parametric. Possible outcome sets were normalised by logarithmic transformation and tested for significance using a parametric method. For categorical variables, an OR was calculated and tested for significance using a Pearson ᵡ² test. P value <0.05 (two-tailed test) was considered to be statistically significant. In addition, all variables underwent a Pearson and Spearman analysis to gauge linear covariance between the outcome measures and the corresponding serum vitamin D concentration.

For binary outcomes, a receiver operating characteristic (ROC) curve was plotted to assess the prognostic value of serum concentrations of vitamin D. Area under the curve (AUC) values >0.5 were deemed to convey a prognostic value in the measured variable.

### Ethics

This survey was approved by the trust audit department with reference FH119 and with clinically collected, non-identifiable data which does not fall under the remit of NHS Research Ethics Committee. All data were collected locally and handled in accordance with European General Data Protection Regulation (GDPR) standards, as well as local and NHS standards on data protection.

All practices conducted as part of this study were done in accordance with local regulations and best clinical practice protocols. Serum vitamin D levels were tested alongside the hospital routine serum COVID-19 panel and did not require any extra phlebotomy. Patients received care in line with standard practices for the management of COVID-19 throughout the study period.

## RESULTS

A total of 105 patients (mean age 81 years, range 65–102; male (n=57):female (n=48)) were recruited to the study, with 70 (66.7%) subsequently allocated to the COVID-19-positive group and 35 (33.3%) allocated to the COVID-19-negative group. Among the COVID-19-positive group, 39 (55.7%) patients were found to have 25(OH)D level ≤30 nmol/L and 31 (44.3%) were found to have a level >30 nmol/L.

Demographics (age, sex, ethnicity, frailty, body mass index, smoking history and comorbidities) between COVID-19-positive and -negative groups were comparable. In addition, the characteristics of vitamin D-replete and -deficient groups in the COVID-19-positive arm were found to be comparable (table 1). Only three patients were admitted from a nursing home with one instance in each of the three study subgroups.

**Table 1:** Population characteristics of COVID-19-positive versus -negative groups, subdivided by serum vitamin D concentrations

Population sample characteristics						
		COVID-19-positive (N=70)			P value	
Demographics		Vitamin D ≤30 nmol/L (N=39)	Vitamin D >30 nmol/L (N=31)	COVID-19-negative (N=35)	COVID-19-positive vit D ≤30 nmol/L vs >30 nmol/L	COVID-19-positive versus COVID-19-egative
Mean age (SD)		79.46 (±9.52)	81.16 (7.23)	83.44 (±8.08)	0.41	0.064
Male:female		24:15	18:13	15:20	0.77	0.075
Rockwood Clinical Frailty Score Median (IQR)		6 (6–7)	5 (5–6)	5 (5–6)	0.1	0.66
Median body mass index (IQR)		25 (23–32)	24 (20–27)	25 (22–29)	0.14	0.75
Smoking status (%)	Current smoker	1 (2.56)	5 (16.13)	4 (11.43)	0.14	0.65
	Ex-smoker	12 (30.77)	11 (35.48)	15 (42.86)	0.66	0.13
Ethnicity (%)	Caucasian	29 (74.36)	21 (67.74)	30 (85.71)	0.51*	0.27*
	South Asian	8 (20.51)	10 (32.36)	3 (8.57)		
	East Asian	2 (5.13)	0 (0)	0 (0)		
	Afro-Caribbean	0 (0)	1 (3.26)	3 (8.57)		
**Comorbidities**		**N (%)**			**P value**	
		**Vitamin D ≤30 nmol/L (N=39)**	**Vitamin D >30 nmol/L (N=31)**	**COVID-19-negative (N=35)**	**COVID-19-positive vit D ≤30 nmol/L vs >30 nmol/L**	**COVID-19-positive versus COVID-19-negative**
Hypertension		18 (46.15)	16 (51.61)	20 (55.56)	0.65	0.41
Diabetes mellitus		17 (43.59)	9 (29.03)	8 (22.22)	0.21	0.14
Ischaemic heart disease		7 (17.95)	8 (25.81)	11 (30.56)	0.43	0.27
Chronic respiratory disease		6 (15.38)	7 (22.58)	4 (11.11)	0.44	0.35
Heart failure		6 (15.38)	6 (19.35)	5 (13.89)	0.66	0.71
Stroke		6 (15.38)	3 (9.68)	3 (8.33)	0.48	0.52
Dementia		4 (10.26)	2 (6.45)	1 (2.78)	0.58	0.29
Chronic kidney disease		10 (25.64)	6 (19.35)	6 (16.67)	0.53	0.5
Atrial fibrillation		6 (15.38)	8 (25.81)	8 (22.22)	0.28	0.73
Cancer		2 (5.13)	1 (3.23)	2 (5.56)	0.7	0.75
Endocrinological disease		1 (7.69)	2 (6.45)	2 (5.56)	0.44	0.75
		**Median value (IQR)**			**P value**	
		**Vitamin D ≤30 nmol/L (N=39)**	**Vitamin D >30 nmol/L (N=31)**	**COVID-19-negative (N=35)**	**COVID-19-positive vit D ≤30 nmol/L vs >30 nmol/L**	**COVID-19-positive versus COVID-19-negative**
Charlson Comorbidity Index		5 (3–6)	4 (4–5)	4 (4–6)	0.81	0.76
		COVID-19-positive (N=70)		COVID-19-negative (N=35	Difference	P value
Vitamin D concentration (nmol/L)		27.00 (20.00–47.00)		52.00 (31.50–71.50)	25	**0.0008**

* P value for ethnicities calculated by comparing the number of Caucasian patients to all other ethnic groups.

vit, vitamin.

No patients were admitted to the intensive treatment unit, and ceilings of care were set to NIV support on the HDU. There was no difference in the average length of stay between vitamin D subgroups (29 days).

Vitamin D levels in the COVID-19-positive group were overall significantly lower compared with that in the COVID-19-negative group (27.00 nmol/L vs 52.00 nmol/L) (p=0.0008). Among patients with vitamin D deficiency in the COVID-19-positive group, there was a higher average peak in D-dimer level (1914.00 μgFEU/L vs 1268.00 μgFEU/L) (p=0.034) and a higher incidence of NIV support and HDU admission (30.77% vs 9.68%) (p=0.042). The vitamin D-deficient case group demonstrated higher peak CRP, LDH and ferritin levels; lower trough lymphocyte counts; and increased incidence of radiographic changes, although these were not statistically significant. The vitamin D-replete case group demonstrated a higher peak troponin level that was not statistically significant (table 2). There was only one confirmed case of pulmonary thrombosis in the vitamin D-deficient case group. There was no apparent difference in mortality between the two groups. Ethnicity did not influence outcomes in this cohort. The outcome measures did not appear to follow a linear relationship with serum vitamin D concentrations.

**Table 2:** Primary and secondary outcome measures, for vitamin D-deficient and -replete groups

Outcome measures				
	Median value (IQR)			
Serum markers	Vitamin D ≤30 nmol/L	Vitamin D >30 nmol/L	Difference	P value
Peak CRP (mg/L)	191.00 (108.00–274.00)	155.00 (96.00–252.00)	−36	0.32
Peak LDH (IU/L)	272.50 (217.25–367.50)	239.50 (180.50–333.00)	−33	0.17*
Peak ferritin (μg/L)	518.50 (894.00–1109.25)	484.50 (221.00–715.50)	−34	0.40*
Peak D-dimer (μgFEU/L)	1914.00 (1323.75–3131.50)	1268.00 (1003.50–2273.00)	−646	**0.034**
Peak troponin(ng/L)	37.00 (26.00–95.00)	42.00 (25.00–88.00)	5	0.83*
Trough lymphocyte count (×10^9^/L)	0.56 (0.44–0.78)	0.68 (0.54–0.95)	0.12	0.15*
	**N (%)**		**OR (CI)**	**P value**
Chest X-ray changes	11 (28.20)	8 (25.80)	1.13 (0.39–3.28)	0.82
Ventilation requirement	12 (30.77)	3 (9.68)	4.15 (1.05–16.34)	**0.042**
Mortality	6 (15.38)	4 (12.90)	1.40 (0.36–5.47)	0.5

* P value derived using a parametric technique on logarithmically transformed data.

CRP, C reactive protein; LDH, lactate dehydrogenase.

**Figure 2:**
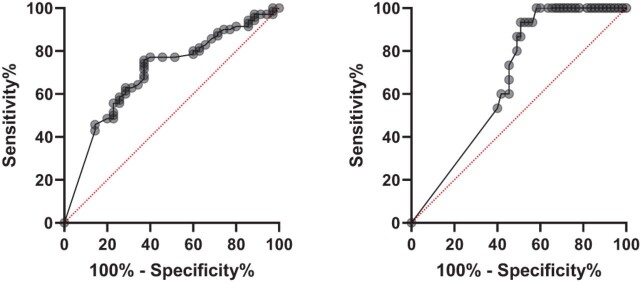
ROC curves for vitamin D and COVID-19 status (left) and ventilatory support requirement (right). ROC, receiver operating characteristic.

ROC curves were plotted for vitamin D as a distinguisher between COVID-19-positive and -negative states as well as between those requiring and not requiring ventilatory support (figure 2). For the former, AUC was 0.70 (95% CI 0.59 to 0.80) (p value=0.0009). For the latter, AUC was 0.67 (95% CI 0.54 to 0.80) (p value=0.046).

## DISCUSSION

The main findings of our study suggest that older patients with lower serum concentrations of 25(OH)D, when compared with aged-matched vitamin D-replete patients, may demonstrate worse outcomes from COVID-19. Markers of cytokine release syndrome were raised in these patients and they were more likely to become hypoxic and require ventilatory support in HDU. There was no difference in mortality between groups.

Evidence of an association between vitamin D deficiency and adverse outcome in COVID-19 is provided by Alipio (2020) and D’Avolio and colleagues.^13^ The former study (preprint) observed an increased disease severity for patients with vitamin D deficiency, while the latter noted a decreased serum vitamin D concentration between COVID-19-positive and -negative patients. These were both retrospective cohort studies encompassing a sample size of 212 and 120, respectively.

Ilie and colleagues^14^ performed a meta-analysis to study the association of vitamin D and morbidity and mortality with COVID-19 in 20 European countries and proposed a possible correlation between vitamin D levels, the incidence of SARS-CoV-2 infection and mortality. Hastie and colleagues^15^ used biobank data on 449 people with confirmed SARS-CoV-2 infection and found no relationship with serum 25(OH)D concentrations. The average age of subjects in this study was 49 years, and a major limitation was utilisation of historical vitamin D levels of patients (between 2006 and 2010) rather than vitamin D status at the time of infection with SARS-CoV-2. In our study, the average age was older at 81 years and we were able to establish vitamin D status during active SARS-CoV-2 infection.

In non-communicable diseases, both viral and bacterial, vitamin D deficiency has been associated with increased morbidity and mortality as well as a higher incidence of acute respiratory distress syndrome in critically unwell patients.^16 17^ Whether low vitamin D levels are cause or consequence of disease (reverse causality) processes remains unclear. However, a meta-analysis performed in 2017 of 11 321 patients from 25 randomised controlled studies demonstrated that vitamin D supplementation protected against acute respiratory tract infection and patients with very low concentrations of 25(OH)D (<25 nmol/L) benefiting most.^18^

Cited markers of cytokine storm were elevated in our vitamin D deficiency subset of patients, and the high peak D-dimer concentration was deemed statistically significant (p=0.034). This was found in the absence of in situ pulmonary thrombosis and in the context of standard VTE prophylaxis. Several studies have explored^19  20^ the pro-thrombotic state induced during the cytokine storm phase of inflammatory lung disease. The coagulation system appears active in critically ill patients, and high D-dimer levels reflect activation of the proinflammatory cytokine cascade (and downregulation of the anti-inflammatory cytokine cascade). Elevated D-dimer levels are associated with amplified risk for multiple organ failure and death.^21^ This may explain the increased incidence of ventilatory support seen in vitamin D-deficient patients.

Vitamin D has been shown to condition the innate immune reaction against both bacterial and viral infections. Calcitriol (1,25(OH)_2_D_3_), the active form of vitamin D, modulates macrophage activity by inhibiting the release of pro-inflammatory cytokines such as interleukin (IL)-1, IL-6, IL-8, IL-12 and tumour necrosis factor-alpha.^16^ Vitamin D shifts the adaptive immune reaction from a Th1 to a Th2 phenotype,^17 22^ downregulating differentiation of naïve T cells into pro-inflammatory Th17 cells,^23 24^ and promotes T regulatory cell induction.^25–27^ Dysregulation of both innate and adaptive immunity, as a result of vitamin D deficiency, may therefore be central to precipitating the ‘cytokine storm’ seen in COVID-19 infection.

In addition to anti-inflammatory properties, vitamin D also exerts a protective effect on human alveolar epithelial cells by promoting wound repair.^28^ Vitamin D has also been shown to preserve endothelial integrity and deficiencies result in increased vascular permeability and leak.^29^

Vitamin D also increases the expression of ACE-2. While increased ACE-2 expression in the early pandemic was predicted to increase the risk of infection, paradoxically, ACE-2 has also been shown to protect against acute lung injury.^14 30^ Disruption of one or more of these defensive pathophysiological processes may explain the association we found between vitamin D deficiency and increased requirement of ventilatory support. Whether this actually represents a causal relationship has not yet been elucidated.

### Limitations

As a single-centre study at a district general hospital, we cannot generalise our results to other settings. Furthermore, our trust is based in Southern England, where population demographics and socioeconomic status may differ from those elsewhere.

We acknowledge that extracting information from medical notes requires second-hand interpretation and may not be representative of the full clinical picture. Patient outcomes may also have been influenced by current guidelines imposed by the National Institute of Clinical Excellence committee.^31^

We also acknowledge the role that sunlight exposure may have played in the measured serum levels of 25(OH)D. Unfortunately, this could not reliably be measured in patients; however, efforts were made to account for this. Notably, the study was carried out within a 2-month period which attenuated potential weather influence on sunlight exposure. In addition, the housing status of patients was considered—only three nursing home residents were included in the study, which minimised the risk of institutionalisation and its association with reduced sun exposure bearing influence on the results.

Considerations were made for the utilisation of sample size calculations to ensure the study was powered appropriately. No limit was initially set for sample size; however, the final number was limited by the dwindling numbers of patients with COVID-19. Despite achieving significant results in several outcomes, we acknowledge the risk of a type 2 error occurring with our experimental sample size. Therefore, we were unable to discount an association between vitamin D deficiency and those variables which did not achieve statistical significance with observed effect sizes.

Length of stay was recorded for all patients in the COVID-19 arm of the study, with no difference found between vitamin D subgroups. We emphasise, however, concerns on the reliability of this measure. Given the geriatric population of our study sample, the overwhelming issues affecting hospital discharge were social and housing issues. This was further complicated by infection control measures during the pandemic limiting the ability of some families to receive their relatives back home. Therefore, any firm conclusions should not be drawn from this outcome measure.

Another issue of consideration was vitamin D replacement and the effect this may have on the outcome measures recorded. In our study, vitamin D supplementation was only initiated after the acute phase of illness. Given that steady-state serum concentrations of vitamin D are achieved after 3–6 months with replacement,^32^ it is unlikely that serum levels would significantly change during the infective and symptomatic phase of admission. Consequently, we do not believe this would have influenced outcome measures. We were also unable to establish whether vitamin D replacement during active SARS-CoV-2 infection results in favourable outcomes.

## CONCLUSION

Our study has demonstrated that patients over the age of 65 years presenting with symptoms consistent with COVID-19 are more likely to be vitamin D deficient. There appears to be a clinically relevant association between this and elevated markers of cytokine release syndrome and increased risk of respiratory failure requiring ventilatory support. Although there was no apparent mortality difference between the two groups, this may reflect the overall poor prognosis associated with the higher prevalence of frailty and comorbidities in our older cohort of patients. Vitamin D status may be a prognosticator for COVID-19, and supplementation might improve outcomes. Further studies in all age groups are awaited to validate this.

**Main messages**

- Older patients with COVID-19 infection and vitamin D deficiency (≤30 nmol/L) have higher peak D-dimer level and higher incidence of NIV support and HDU admission.
- Vitamin D deficiency may be associated with worse outcomes from COVID-19, and vitamin D status may be a useful prognosticator.

**What is already known on the subject**

- There appears to be an association between increased COVID-19 incidence and mortality and countries with an increased prevalence of vitamin D deficiency.
- Vitamin D plays an important role in the modulation of the immune system through promotion of anti-inflammatory cytokines and down-regulation of pro-inflammatory T cells.
- The occurrence of an inflammatory ‘cytokine storm’ during COVID-19 has been associated with poorer outcomes and increased disease severity.
